# Supplementary material for: Application of Thermally Fluorinated Multi-Wall Carbon Nanotubes as an Additive to an Li4Ti5O12 Lithium Ion Battery
Source: Nanomaterials (Basel). 2023 Mar 9;13(6):995. doi: 10.3390/nano13060995 (PMC10059772; doi:10.3390/nano13060995)
Supplement: Supplementary file 1 [file nanomaterials-13-00995-s001.zip › nanomaterials-2228808-supplementary.docx]

Supplementary Materials

Application of Thermally Fluorinated Multi-Wall Carbon Nanotubes as an Additive to an Li_4_Ti_5_O_12_ Lithium Ion Battery

Seongmin Ha ^1^, Seo Gyeong Jeong ^1^, Chaehun Lim ^1^, Chung Gi Min ^1^ and Young-Seak Lee ^1,2,^*

^1^ Department of Chemical Engineering and Applied Chemistry, Chungnam National University,
Daejeon 34134, Republic of Korea; haseongmin93@cnu.ac.kr (S.H.)

^2^ Institute of Carbon Fusion Technology (InCFT), Chungnam National University,
Daejeon 34134, Republic of Korea

***** Correspondence: youngslee@cnu.ac.kr

**Figure S1.** X-ray diffraction (XRD) patterns of MWCNTs, F-MWCNTs_6, and F-MWCNTs_12.


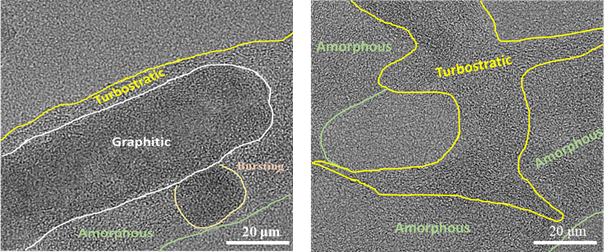


**Figure S2.** TEM images of MWCNTs, F-MWCNTs_6, and F-MWCNTs_12.


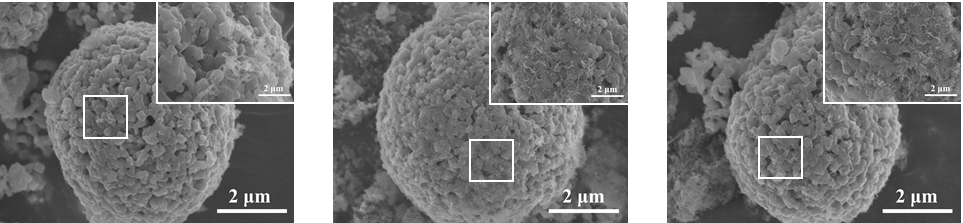


**Figure S3.** SEM images of MWCNTs, F-MWCNTs_6, and F-MWCNTs_12.


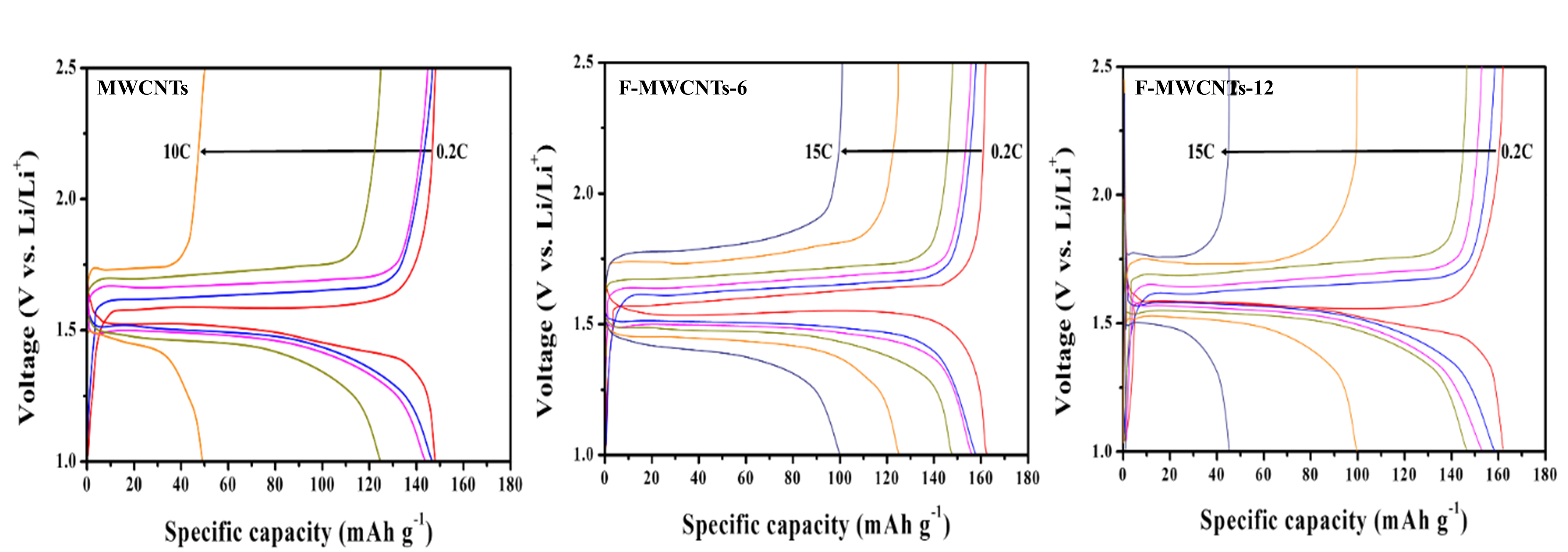


**Figure S4.** Galvanostatic charge/discharge curves of MWCNTs, F-MWCNTs_6, and F-MWCNTs_12 at various rates (0.2, 1, 2, 5, 10, 15 C).
