# Supplementary material for: Application of Thermally Fluorinated Multi-Wall Carbon Nanotubes as an Additive to an Li4Ti5O12 Lithium Ion Battery
Source: Nanomaterials (Basel). 2023 Mar 9;13(6):995. doi: 10.3390/nano13060995 (PMC10059772; doi:10.3390/nano13060995)
Supplement: Supplementary file 1 [file nanomaterials-13-00995-s001.zip › nanomaterials-2228808-supplementary.pdf]

nanomaterials

Supplementary Materials

Application of Thermally Fluorinated Multi-Wall Carbon Nanotubes as an Additive to an $Li_4Ti_5O_{12}$ Lithium Ion Battery

Seongmin Ha [1], Seo Gyeong Jeong [1], Chaehun Lim [1], Chung Gi Min [1] and Young-Seak Lee [1,2,*]

[1] Department of Chemical Engineering and Applied Chemistry, Chungnam National University, Daejeon 34134, Republic of Korea; haseongmin93@cnu.ac.kr (S.H.)

[2] Institute of Carbon Fusion Technology (InCFT), Chungnam National University, Daejeon 34134, Republic of Korea

* Correspondence: youngslee@cnu.ac.kr

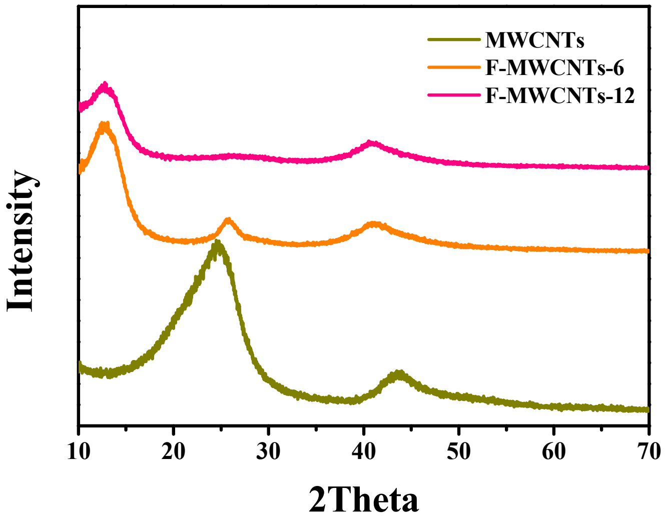

Figure S1. X-ray diffraction (XRD) patterns of MWCNTs, F-MWCNTs_6, and F-MWCNTs_12.

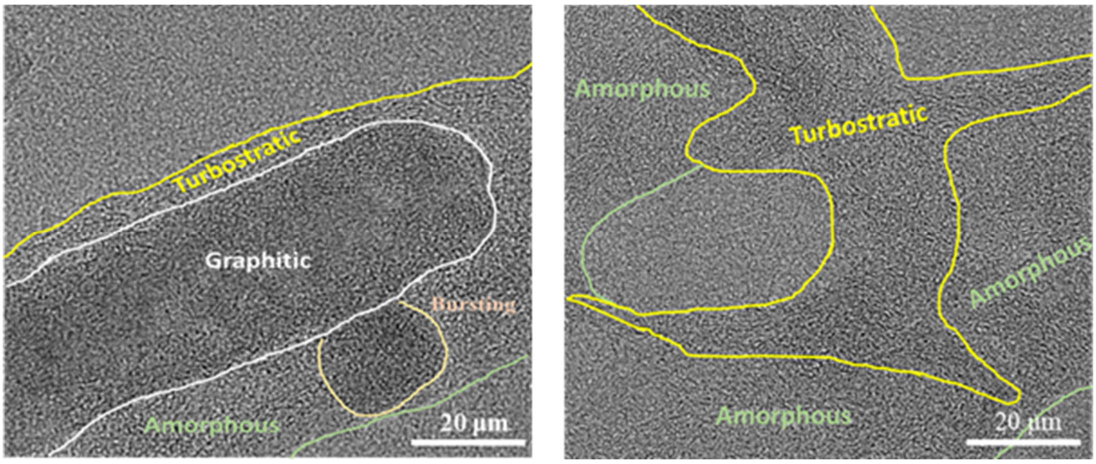

Figure S2. TEM images of MWCNTs, F-MWCNTs_6, and F-MWCNTs_12.

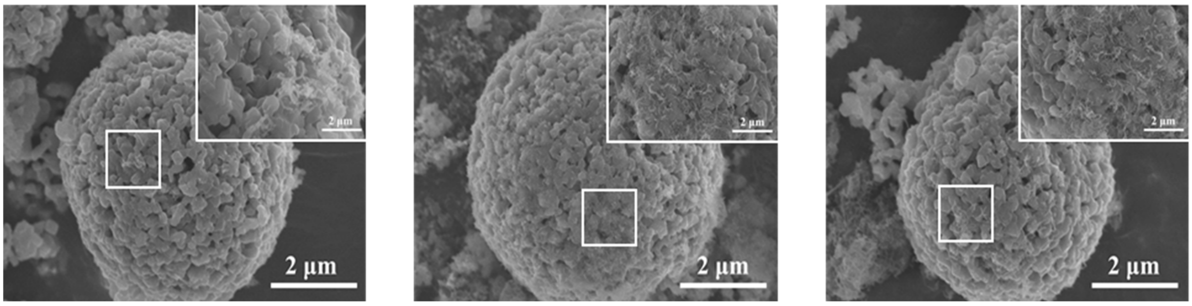

Figure S3. SEM images of MWCNTs, F-MWCNTs_6, and F-MWCNTs_12.

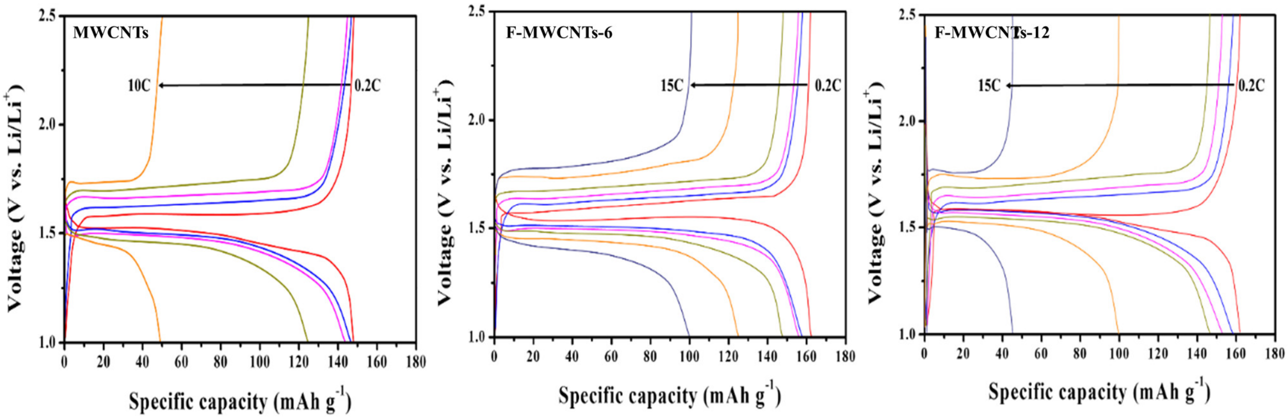

Figure S4. Galvanostatic charge/discharge curves of MWCNTs, F-MWCNTs_6, and F-MWCNTs_12 at various rates (0.2, 1, 2, 5, 10, 15 C).
